# The eternal quest for self-improvement of somatic cells

**DOI:** 10.1016/j.xgen.2022.100094

**Published:** 2022-02-09

**Authors:** Peter J. Campbell

**Affiliations:** 1 Wellcome Sanger Institute, Hinxton CB10 1SA, UK

## Abstract

Multiple somatic mutations drive cancer cell transformation, but the sequence of events is poorly understood. In this issue of *Cell Genomics*, Kester et al. combine organoid technology, prospective barcode labeling, and single-cell sequencing to study the evolution of somatic clones *in vitro*.

## Teaser

Multiple somatic mutations drive cancer cell transformation, but the sequence of events is poorly understood. In this issue of *Cell Genomics*, Kester et al. (2022) combine organoid technology, prospective barcode labeling, and single-cell sequencing to study the evolution of somatic clones *in vitro*.

## Main text

Cancer arises through the acquisition of mutations that confer growth potential and other malignant properties on a somatic clone. Most cancers require multiple, perhaps 5–20, somatic mutations to fully transform the cell ^1^. These so-called “driver mutations” can range from single base substitutions through structural variants to whole chromosome gains and losses. While genome sequencing studies have systematically identified the driver mutations that cause human cancers, they have provided only partial insights into the temporal dynamics of cancer evolution. Sequencing an incident cancer from a patient provides only a retrospective view of that evolution—we only observe the winners of the Darwinian competition, meaning that it can be difficult to infer the order in which driver mutations occurred. In this issue of *Cell Genomics*, Kester et al. ^2^ develop fascinating methods to study evolution of somatic clones prospectively *in vitro*.

All methods for characterizing clonal evolution in somatic cells have important limitations and drawbacks. Bulk genome sequencing lacks sensitivity for all but the most prevalent subclones; copy number aberrations (CNAs) can be robustly identified from single-cell sequencing, but inferring clonally related CNAs is challenging due to low numbers of events and frequent parallel evolution; base substitutions from single-cell sequencing are subject to numerous artifacts of whole-genome amplification; and barcode labeling only marks the subclones at one point in time, therefore lacking the temporal resolution of spontaneous somatic mutations.

The solution to these methodological limitations that Kester et al. ^2^ deployed was to use all assays in combination on the same long-term organoid culture of human colonic epithelium. The organoid was engineered using CRISPR-Cas9 to have knockouts of *APC*, *TP53*, and *SMAD4* together with knockin of *KRAS*^G12D^—the commonest and earliest driver mutations seen in colorectal carcinoma ^3^^,^^4^. They labeled the organoid cells using a library of 60,000 viral barcodes at the outset and set the labeled cells off on their 6-month evolutionary journey. Sampling the cultures weekly, they quantified the barcode composition to track clones at high temporal resolution. These data were further enhanced with CNA data from 3–5 single-cell-sequencing experiments per replicate spread across the 6 months and base substitution data from combined bulk and single-cell sequencing. Importantly, data from these various methods were combined to provide an accurate, integrated picture of the organoid’s clonal dynamics over the 6 months, combining information on both the size and recurrent CNAs present in each subclone.

Intriguing patterns emerged. The sequential barcode data revealed an evolutionary dynamic shaped more by positive than negative selection. The richly polyclonal landscape of the culture was maintained for many months, with the vast majority of those clones showing stable maintenance of low-level cellular fractions over that time (see Figure S1 in Kester et al.^2^). A few clones, though, underwent steady expansion throughout the experiment, resulting in oligoclonality coming to dominate the cellular mix by the last month or two. This dynamic in which the vast majority of clones are in stable equilibrium but gradually out-competed by a minority of clones is more consistent with the minority clones having an additional acquired selective advantage (positive selection) rather than the majority all having additional acquired disadvantage (negative selection). This dominance of positive selection mirrors that seen in somatic cells *in vivo*, both in cancers ^5^^,^^6^ and normal tissues ^7^^,^^8^—germline genomes, in contrast, are much more strongly shaped by negative selection.

Of course, with the genomes sequenced as well as the barcodes, Kester et al. ^2^ could then examine what genomic changes underpinned the selective advantage of the subclones that expanded. A particularly striking finding was that loss of chromosome 18 occurred in several subclones within the experiment. Furthermore, in several clones with loss of 18, chromosome 4 was subsequently lost, with barcode and base substitution data suggesting that these were independently acquired deletions—an example of parallel evolution. The fastest growing clone across the replicates was a clone that lost chromosome 18 and then chromosome 4, and the authors show that this order of events may be a frequent pattern in patient tumors. The appealing hypothesis to emerge out of these data is that chromosome 4 loss only confers a selective advantage when chromosome 18 is already lost, an example of epistasis operating at the level of whole chromosome aneuploidies. Cancers in which the order of mutation acquisition matters have been described for point mutations ^9^; however, it has been less well established for copy number changes, although it is known that recurrent patterns of losses and gains are observed across multiple chromosomes in many solid tumors ^10^.

In their eternal quest for self-improvement, somatic cells explore a wide-ranging portfolio of genomes. Even when already loaded with four hard-hitting driver mutations, their genomes can be further fine-tuned to enhance growth in a specific environment. The early driver mutations likely shape the set of subsequent mutations that confer selective advantage, bringing a predictability to the trajectories of cancer evolution that can now be documented *in vitro* and *in vivo*.

## References

[1] Vogelstein B., Papadopoulos N., Velculescu V.E., Zhou S., Diaz L.A., Kinzler K.W. (2013). Cancer genome landscapes. Science.

[2] Kester L., de Barbanson B., Chen L., Lyubimova A., van der Schrier V., Alemany A., Mooijman D., Peterson J., Drost J., de Ridder J., van Oudenaarden A. (2022). Integration of multiple lineage measurements from the same cell reconstructs parallel tumor evolution. Cell Genomics.

[3] Cancer Genome Atlas Network (2012). Comprehensive molecular characterization of human colon and rectal cancer. Nature.

[4] Gerstung M., Jolly C., Leshchiner I., Dentro S.C., Gonzalez S., Rosebrock D., Mitchell T.J., Rubanova Y., Anur P., Yu K., PCAWG Evolution & Heterogeneity Working Group, PCAWG Consortium (2020). The evolutionary history of 2,658 cancers. Nature.

[5] Martincorena I., Raine K.M., Gerstung M., Dawson K.J., Haase K., Van Loo P., Davies H., Stratton M.R., Campbell P.J. (2017). Universal Patterns of Selection in Cancer and Somatic Tissues. Cell.

[6] Weghorn D., Sunyaev S. (2017). Bayesian inference of negative and positive selection in human cancers. Nat. Genet..

[7] Martincorena I., Roshan A., Gerstung M., Ellis P., Van Loo P., McLaren S., Wedge D.C., Fullam A., Alexandrov L.B., Tubio J.M. (2015). Tumor evolution. High burden and pervasive positive selection of somatic mutations in normal human skin. Science.

[8] Ng S.W.K., Rouhani F.J., Brunner S.F., Brzozowska N., Aitken S.J., Yang M., Abascal F., Moore L., Nikitopoulou E., Chappell L. (2021). Convergent somatic mutations in metabolism genes in chronic liver disease. Nature.

[9] Ortmann C.A., Kent D.G., Nangalia J., Silber Y., Wedge D.C., Grinfeld J., Baxter E.J., Massie C.E., Papaemmanuil E., Menon S. (2015). Effect of mutation order on myeloproliferative neoplasms. N. Engl. J. Med..

[10] Davoli T., Xu A.W., Mengwasser K.E., Sack L.M., Yoon J.C., Park P.J., Elledge S.J. (2013). Cumulative haploinsufficiency and triplosensitivity drive aneuploidy patterns and shape the cancer genome. Cell.

